# Staying in the game: protective and risk factors for burnout in soccer coaching

**DOI:** 10.3389/fpsyg.2025.1617521

**Published:** 2025-09-16

**Authors:** Karol Wasielewski, Andrzej Szwarc, Dominika Maria Wilczyńska

**Affiliations:** ^1^Faculty of Physical Education, Gdansk University of Physical Education and Sport, Gdańsk, Poland; ^2^Faculty of Social and Humanities, WSB Merito University in Gdansk, Gdańsk, Poland

**Keywords:** professional burnout, soccer coaches, predictors, personal development, coaching experience

## Abstract

**Purpose:**

Burnout is a noticeable problem both in education and in sports. Soccer coach operates in both. The purpose of this study was to investigate how age, coaching experience, education, professional qualifications and professional development -interact to predict burnout among soccer coaches.

**Methods:**

The study sample consisted of 412 participants. Coaches were asked to complete the Soccer Coach Questionnaire, which consists of the following sections: demographic variables, personal development, coaching experience, and satisfaction with salary. Afterwards, they were asked to fill out Link Burnout Questionnaire (LBQ). Statistical analysis was performed in the Automated Statistical Description System (SZTOS).

**Results:**

Age was found to be negatively associated with soccer coaches’ burnout, particularly among those motivated by earning points for license renewal. Also, it was showed that coaching experience is inversely associated with coaches’ burnout, but primarily among those coaches who were motivated by earning education points for license renewal. The satisfaction with salary did not moderate any of the association with measured variables as the authors expected. Among coaches motivated by earning points for license renewal, age was found to be negatively correlated with burnout (*p* = 0.006). Similarly, coaching experience was shown to be inversely correlated with burnout among coaches motivated by earning points for license renewal (*p* = 0.008). The effect sizes limit the generalizability of findings. The satisfaction with salary did not moderate any of the association with measured variables as the authors expected.

**Conclusion:**

The results suggest that professional burnout may affect younger and less experienced coaches to a greater extent particularly in the context of retraining. Along with age and experience, the rigor of scoring positively affects coaches which suggests a variable pathway of retraining depending on these factors. A greater number of led teams has a positive effect on burnout but not in coaches with higher specialist training suggesting their need for self- actualization and fulfilment. The authors suggest a further need for research into professional burnout in soccer coaches.

## Introduction

Professional burnout is sometimes described as an “illness of over commitment,” which most often arises when values, goals, and the mental and physical costs of the job are compared. Burnout is not simply fatigue, as a person can be very tired from work, but derive satisfaction from it. It is most often suggested that the process of burnout in the profession begins very slowly and imperceptibly, and reveals itself suddenly and with great force (Siwiorek, 2018). There are numerous models of professional burnout and so far, no single universal model of occupational burnout syndrome has emerged, but rather various theoretical approaches. Currently, the dominant concept of burnout is the triadic symptom model created by Maslach and Jackson (1981), which comprises (1) emotional exhaustion, (2) depersonalization or a tendency toward cynicism or lack of empathy and (3) decreased personal accomplishment (Parker et al., 2023). However, Santinello created a new model of professional burnout where the researcher rephrased three dimensions based on Maslach and Jackson model to get dimensions most consistent with theoretical definitions. Santinello expanded his model by adding one new dimension, that of disillusionment, to the three traditionally considered, yielding a final four dimensions of occupational burnout: (1) psychophysical exhaustions, (2) impaired relations, (3) professional inefficacy and (4) disillusion (Santinello, 2007, as cited in Jaworowska, 2014).

Occupational burnout is a common phenomenon that affects various social and professional groups, especially those professions that involve extensive helping and interpersonal contact. Therefore, the problem of burnout largely affects education-related professions in the broadest sense. Research indicates that teachers are often at risk of professional burnout, which can lead to a deterioration in the learning outcomes of pupils in their charge and, as a consequence, a deterioration in educational outcomes in schools (Agyapong et al., 2023; Capone et al., 2019; Iancu et al., 2018; Madigan and Kim, 2021). A coach is a special kind of educator in a particular discipline. The problem of burnout also occurs in this profession and affects many coaches in many disciplines (Gencay and Gencay, 2011; Hudson et al., 2013; Seo et al., 2022). Soccer coaches, especially those working in the top leagues, experience symptoms of burnout such as chronic fatigue, depersonalization or decreased job satisfaction, often due to a lack of support from clubs and the pressure of performance responsibility (Gustafsson et al., 2011; Hassmén et al., 2019; Hjälm et al., 2007; Koustelios, 2010; Lundkvist et al., 2012). In the long term, this can lead to a deterioration of relationships with players, their own burnout and a decrease in satisfaction with training (Jiahao and Jing, 2024).

The mentioned models capture the structural features of occupational burnout, but they provide limited insight into the motivational mechanisms that may contribute to its development or prevention. To address this gap, Self-Determination Theory (SDT) (Deci and Ryan, 1985; Ryan and Deci, 2000, 2017) offers a comprehensive framework for understanding the motivational foundations of burnout and wellbeing. Burnout among educators, especially among coaches, can arise from a variety of factors. The wide range of theoretical approaches to burnout reflects the complexity of the phenomenon, which can be analyzed at multiple levels: individual, interpersonal, and organizational. One key factor contributing to burnout may be the lack of opportunities to fulfill fundamental psychological needs in the workplace. According to SDT, individuals experience greater life satisfaction and enhanced psychological wellbeing when their needs for autonomy, competence, and relatedness are satisfied. This theory can be applied across domains—including work, education, and sport—and provides a broad framework for studying human motivation. This theory can be universally applied to many domains including work, education, sport and is a broad framework to study human motivation. There are observations from literature which prove that when environment supports the individual’s experience of competency, relatedness and autonomy it can foster motivation and work engagement which in turn will improve performance, energy, resilience and creativity and decrease levels of burnout (Moen et al., 2018; Poulsen and Poulsen, 2018; Shi, 2024). These factors should be addressed at a association level, club and a personal level if the best outcomes are to be achieved.

### Aim of the study

**Figure 1:**
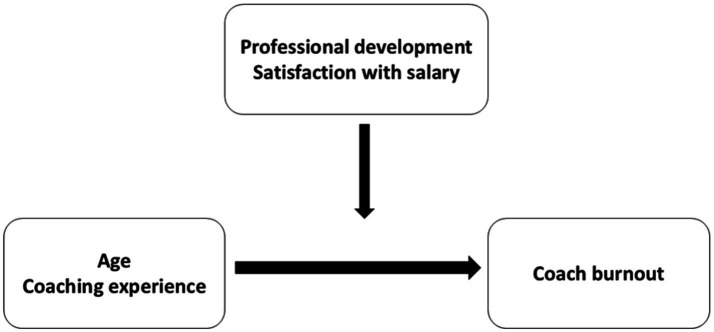
Model of predictors of professional burnout of soccer coaches.

Despite many studies on burnout among educators and coaches, significant gaps remain-particularly regarding burnout among soccer coaches working with amateur and youth teams. For instance, burnout in elite soccer coaches has been depicted, with Swedish data showing that 71% of women’s league coaches and 23% of men’s league coaches experienced moderate to high emotional exhaustion (Hjälm et al., 2007). Similarly, qualitative work with elite coaches in Sweden has linked burnout to work-home conflict and inadequate recovery (Olusoga et al., 2019). However, little is known about burnout in coaches leading amateur or youth soccer, who often face limited support, varied workloads, and diverse coaching qualifications. It is also unclear how coaching experience, professional development, and workload differences contribute to risk—or potentially buffer against burnout. Addressing these gaps is essential for informing tailored strategies to support coach wellbeing across different settings. The authors in the following article attempted to explore these relationships. To this end, an attempt was made to analyze the model, which is shown in Figure 1.

Taking into account the model above the three hypotheses were created as below:

*H1:* Age is associated with coaches’ burnout level and this relation is moderated by selected aspects of professional development and satisfaction with salary. From an SDT perspective, these contextual factors may influence the extent to which coaches experience competence, autonomy, and relatedness in their work, thereby strengthening or buffering the impact of age on burnout.

*H2:* Coaching experience is associated with coaches’ burnout and this relation is moderated by selected aspects of professional development and satisfaction with salary. Consistent with SDT, greater experience may enhance perceived competence and autonomy, but if professional growth or financial recognition are lacking, these needs may be frustrated, increasing the risk of burnout.

Although the present study did not directly measure basic psychological needs, variables such as age, coaching experience, professional development, and salary satisfaction can be understood as contextual factors that may shape the degree to which these needs are met.

## Materials and methods

### Study design

The study involved licensed soccer coaches who were participants in a training conference organized by the Polish Football Association. All participants held UEFA coaching licenses at the time of study. In both the conference and the survey, participation was voluntary and anonymous. The participants were informed about the purpose of the study and the procedure for its conduct. The respondents completed the survey questionnaire after the conference under identical conditions and with no time limit. Data were collected using a dedicated Microsoft Forms survey, created specificity for the purpose of the study. The questionnaire was accessible only during the data collection period involving the study participants. The study received ethical approval from Ethics Board for Research Projects at the University of Physical Education and Sport in Gdansk.

### Participants

The study sample consisted of 412 participants. Regarding gender, the majority identified as male (*n =* 386, 93.69%), while a small proportion identified as female (*n =* 26, 6.31%). In terms of geographical distribution, most participants were from the Pomeranian Voivodeship (*n =* 395, 95.87%), with a minority from other regions (*n =* 17, 4.13%). Participants reported various places of residence: cities (*n =* 142, 34.47%), towns (*n =* 113, 27.43%), villages (*n =* 105, 25.49%), and small cities (*n =* 52, 12.62%). With respect to education, 169 participants (41.02%) held a higher education degree in sports, 141 (34.22%) had primary or secondary education, and 102 (24.76%) reported higher education in fields other than sports. Concerning coaching qualifications, 143 participants (34.71%) held a UEFA B license, 139 (33.74%) had a UEFA C or GRASSROOTS C license, and 130 (31.55%) were qualified at the UEFA A level. Table 1 shows descriptive statistics for numerical variables.

**Table 1:** Descriptive statistics.

Variable	*N*	*Min*	*Max*	*M*	*SD*	*SE*	*Me*
Age	412	18	72	38.83	10.65	0.52	39
Coaching experience	412	0	44	11.17	8.62	0.42	10
Number of teams managed	412	0	3	1.25	0.86	0.04	1
Coach burnout score (LBQ)	412	4.3	16.69	8.95	2.67	0.13	8.64

*N*, Sample size; *Min,* Minimum value; *Max*, Maximum value; *M*, Mean; *SD*, Standard deviation; *SE*, Standard error of the mean; *Me*, Median.

### Procedure

Coaches were given instructions on how to complete the survey. They were then given access to an online survey, which they could complete on any device with internet access. The survey was completed without interference from the authors and with no time limit. All participants provided informed consent prior to the study, and their data were anonymized to ensure confidentiality. The study received approval from the Ethics Board for Research Projects at the University of Physical Education and Sport, Gdansk, Poland (Resolution number 1/15.07.2024).

### Measures

Soccer Coach Questionnaire—the questionnaire is prepared for the purpose of this study and is divided into four parts which describe and investigate different aspects. The sections were named accordingly:

1. *Demographics*: gender, date of birth, voivodeship of residence, place of residence
2. *Personal development*: education level, coaching qualifications, and eight questions as follow: Do you deepen your knowledge through in-person workshops, conferences, or trainings? Why do you attend in-person conferences/trainings/workshops? Do you participate in webinar-based trainings? Why do you prefer webinar-based trainings? How many training credits do you earn on average over 3 years? Where do you mostly earn your training credits? In your opinion, what is particularly important to achieve success as a sports coach?
3. *Coaching experience* consists of seven questions: What is your coaching experience (in years)? What is the highest level of soccer you played? How many teams do you currently coach? What is your role in the club? How many training sessions do you conduct weekly (excluding matches or tournaments)? On average, how many days per week do you coach? Where you currently work as coach?
4. *Satisfaction with salary* with two questions: s your current coaching salary satisfactory and does it meet your expectations? Does your salary meet your expectations in terms of responsibilities (teams, sessions, additional functions)?

The questionnaire varies in terms of possible answers and does not have regular Likert scale, therefore to get more information please contact the first author. In the current study, the questionnaire is undergoing its first pilot test.

### LBQ: link burnout questionnaire

This is a self-report questionnaire which provides new burnout indicators for adults who work in different professions including education, health service, sport and many others. The questionnaire was created by Santinello (2007) and adapted to polish conditions by Jaworowska (2014). The LBQ consists of 24 items with a six-point Likert-type response scale for study of four dimensions, each with three positive and three negative elements: the psychophysical dimension (energy-exhaustion), relationships (involvement-deterioration), professional competence (efficacy-inefficacy), and existential expectations (satisfaction-disillusion). Within the scope of this study, the Cronbach’s alpha for the complete questionnaire was *α* = 0.85. For the purpose of this study, we examined the general score for the burnout of the investigated coaches.

### Statistical analysis

This analysis was performed in the Automated Statistical Description System - SZTOS (Hryniewicz and Milewska, 2023). The results were visualized using the graphics package “ggplot2” (Wickham, 2016). The SZTOS application is software developed by the Polish company SZTOS. The software is written in R language and its distinctive feature is the generation of comprehensive descriptions of results and drawings based on logical decision processes. Effect sizes were reported accordingly to Cohen guidelines (Cohen, 1988). Preliminary data exploration showed that missing values accounted for 1.63% of the entire dataset. They were filled using the “missForests” method (Stekhoven and Bühlmann, 2012), implemented in the SZTOS program. This is a nonparametric method that uses random forests to maximize the prediction of values at missing data locations (Breiman, 2001). To analyze the results in terms of LBQ burnout scores, an analysis of variance (Fisher, 1921) was conducted with two main effects and its interaction. The analysis of variance used type III sum of squares, which means that each variable (main or interaction effect) is evaluated taking into account all others. Furthermore due to the non-normal distribution of the LBQ score, this variable was subjected to a normalization procedure. To select the best method for normalizing the LBQ score variable, a series of transformations of this variable were performed using a range of normalization methods. The R package bestNormalize (Peterson, 2021) was used for this purpose implemented in the SZTOS program. The analysis showed that the orderNorm technique yielded the best normalization properties. Compared to other popular transformations, this method returned the lowest score of fit statistics value, P/df = 0.01. Visual diagnostics of the tested models in terms of posterior predictive values, variance homogeneity, collinearity outliers, and normality of residuals performed in the performance package (Lüdecke et al., 2021) showed that models with normalized LBQ scores were closer to meeting the assumptions of the analysis of variance model than raw LBQ scores. However, the diagnostics revealed that the model’s linearity assumption was not met and that the multicollinearity statistics for the main effects were slightly elevated. However, this is not a cause for concern when testing models containing interaction components resulting from simultaneous testing of main effects. Additionally, formal comparative analyses of the models showed that models with normalized values of the LBQ variable were significantly and significantly better fitted to the data than models tested on non-normalized data. Results section includes tables comparing model performance, and diagnostic plots are shown both before and after LBQ normalization. All results reported below are expressed in results after normalization and standardized.

## Results

### *Analysis 1*: age and motivation for education as earning points (personal development- why do you attend in-person conferences/trainings/workshops?)

**Figure 2:**
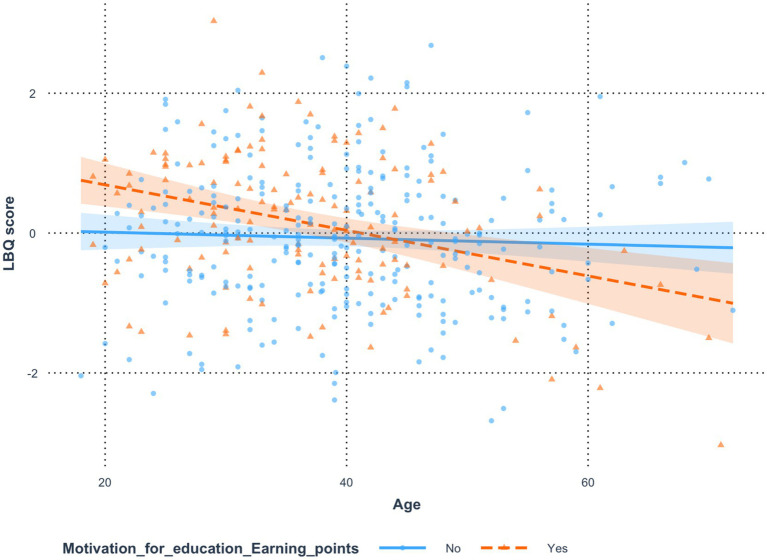
Relation between age and burnout moderated by motivation for education as earning points (personal Development- Why do you attend in-person conferences/trainings/workshops?).

To analyze the results, an analysis of variance (Fisher, 1921) was conducted, which showed that the variable Age did not significantly influence LBQ score [*F*(1, 408) = 0.59, *p* = 0.444, *η*^2^ = 0.00, $\eta_{p}^{2}$ = 0.00]. In contrast, the variable Motivation for Education: Earning Points had a significant effect [*F*(1, 408) = 10.62, *p* = 0.001, *η*^2^ = 0.02, $\eta_{p}^{2}$ = 0.03]. Furthermore, the analysis of interaction effects in the tested model revealed a significant interaction between Age and Motivation for Education: Earning Points in predicting LBQ score [*F*(1, 408) = 8.47, *p* = 0.004, *η*^2^ = 0.02, $\eta_{p}^{2}$ = 0.02]. The analysis of the model’s R^2^ coefficient and its adjusted value indicated that the model explained approximately 5.09% (adjusted: 4.39%) of the variance in LBQ score. Figure 2 shows that the relationship between Age and LBQ score differed between the “Points-Motivated” and “Non-Points-Motivated” groups. The magnitude of this difference was small, with Cohen’s *d* = 0.03, 95% CI [0.01, 0.05]. In the Non-Points-Motivated group, Age was not significantly related to LBQ score, *b* = 0.00, *t*(408) = −0.77, *p* = 0.444. In contrast, in the Points-Motivated group, higher Age was significantly associated with lower LBQ score, *b* = −0.03, *t*(408) = −4.10, *p* < 0.001 (Tables 2, 3).

**Table 2:** Estimates of the significance of differences in the effect of the variable *age* on the variable *LBQ score* resulting from the moderating effect of the variable *motivation for education—earning points.*

Comparison	Difference	*t*	*p*	Cohen’s d	LCI	UCI
No*—*Yes	0.03	2.91	0.004	0.03	0.01	0.05

Compariso*n =* Statistical assessment of differences in the effect of the variable *Age* on the variable *LBQ score* between groups of the moderating variable *Motivation for education—Earning points*. Difference = Magnitude of the difference in score levels between the respective groups of the variable *Motivation for education—Earning points*. t = Student’s t statistic. Cohen’s d = Effect size coefficient *d* by Cohen. LCI = Lower confidence interval for Cohen’s *d*. UCI = Upper confidence interval for Cohen’s *d*. p = Statistical significance calculated without correction for pairwise multiple comparisons.

**Table 3:** Regression estimates of the effect of the variable *age* on the variable *LBQ score* in subgroups of the moderating variable *motivation for education—earning points.*

Group	b	s.e.	LCI	UCI	*t*	*p*
No	0.00	0.01	−0.02	0.01	−0.77	0.444
Yes	−0.03	0.01	−0.05	−0.02	−4.10	< 0.001

b = Estimate of the effect of the variable *Age* on the variable *LBQ score* resulting from the moderating effect of the variable *Motivation for education—Earning points*. s.e. = Standard error of the estimate *b*. t = Student’s t statistic. LCI = Lower confidence interval for *b*. UCI = Upper confidence interval for *b*. p = Statistical significance calculated without correction for pairwise multiple comparisons.

### *Analysis 2*: age and coach qualifications (personal development)

**Figure 3:**
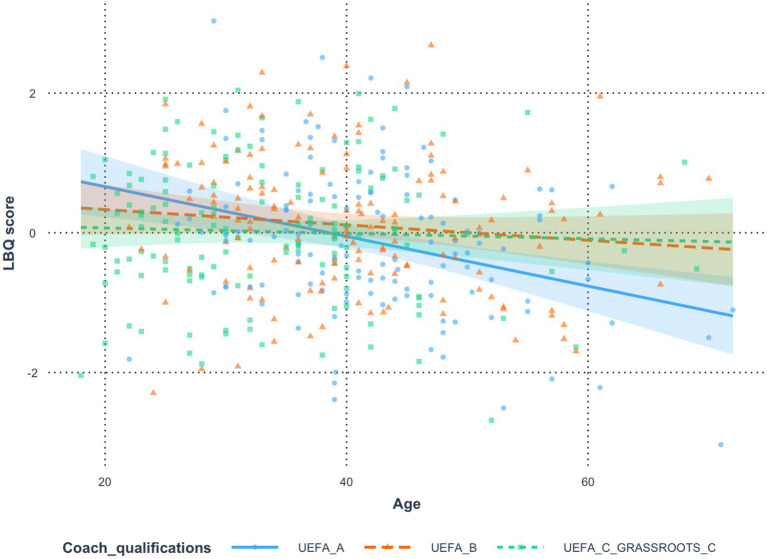
Relation between age and burnout moderated by motivation for education as earning points (personal development- why do you attend in-person conferences/trainings/workshops?).

To analyze the results, an analysis of variance (Fisher, 1921) was conducted, which showed that Age had a significant effect on LBQ score [*F*(1, 406) = 15.22, *p* < 0.001, *η*^2^ = 0.04, $\eta_{p}^{2}$ = 0.04], as did Coach qualifications [*F*(2, 406) = 3.12, *p* = 0.045, *η*^2^ = 0.01, $\eta_{p}^{2}$ = 0.02]. Furthermore, the analysis of interaction effects in the tested model revealed a significant interaction between Age and Coach qualifications in predicting LBQ score [*F*(2, 406) = 3.60, *p* = 0.028, *η*^2^ = 0.02, $\eta_{p}^{2}$ = 0.02]. The model explained approximately 5.08% of the variance in LBQ score (adjusted: 3.91%). Figure 3 shows that the effect of Age on LBQ score differed between qualification groups. The difference between UEFA A and UEFA B was small, *b* = −0.02, *t*(406) = −2.03, *p* = 0.043, Cohen’s *d* = −0.03, 95% CI [−0.05, 0.00]. In the UEFA A group, higher Age was significantly associated with lower LBQ score (*b* = −0.04, *t* = −3.90, *p* < 0.001), whereas in the UEFA B group, this relationship was not statistically significant (*b* = −0.01, *t* = −1.39, *p* = 0.166). The difference between UEFA A and UEFA C GRASSROOTS C was also small, *b* = −0.03, *t*(406) = −2.59, *p* = 0.010, Cohen’s *d* = −0.03, 95% CI [−0.06, −0.01]. In the UEFA C GRASSROOTS C group, the relationship between Age and LBQ score was not significant (*b* = 0.00, *t* = −0.48, *p* = 0.635). No significant difference was found between UEFA B and UEFA C GRASSROOTS C, *b* = −0.01, *t*(406) = −0.63, *p* = 0.532, Cohen’s *d* = −0.01, 95% CI [−0.03, 0.02] (Tables 4, 5 and Figure 3).

**Table 4:** Estimates of the significance of differences in the effect of the variable *age* on the variable *LBQ score* resulting from the moderating effect of the variable *coach qualifications.*

Comparison	Difference	*t*	*p*	Cohen’s *d*	LCI	UCI
UEFA A*—*UEFA B	−0.02	−2.03	0.043	−0.03	−0.05	0.00
UEFA A*—*UEFA C GRASSROOTS C	−0.03	−2.59	0.010	−0.03	−0.06	−0.01
UEFA B*—*UEFA C GRASSROOTS C	−0.01	−0.63	0.532	−0.01	−0.03	0.02

Compariso*n =* Statistical assessment of differences in the effect of the variable *Age* on the variable *LBQ score* between groups of the moderating variable *Coach qualifications*. Difference = Magnitude of the difference in score levels between the respective groups of the variable *Coach qualifications*. t = Student’s t statistic. Cohen’s d = Effect size coefficient *d* by Cohen. LCI = Lower confidence interval for Cohen’s *d*. UCI = Upper confidence interval for Cohen’s *d*. p = Statistical significance calculated without correction for pairwise multiple comparisons.

**Table 5:** Regression estimates of the effect of the variable *age* on the variable *LBQ score* in subgroups of the moderating variable *coach qualifications.*

Group	b	s.e.	LCI	UCI	*t*	*p*
UEFA A	−0.04	0.01	−0.05	−0.02	−3.90	< 0.001
UEFA B	−0.01	0.01	−0.03	0.00	−1.39	0.166
UEFA C GRASSROOTS C	0.00	0.01	−0.02	0.01	−0.48	0.635

b = Estimate of the effect of the variable *Age* on the variable *LBQ score* resulting from the moderating effect of the variable *Coach qualifications*. s.e. = Standard error of the estimate *b*. t = Student’s t statistic. LCI = Lower confidence interval for *b*. UCI = Upper confidence interval for *b*. p = Statistical significance calculated without correction for pairwise multiple comparisons.

### *Analysis 3*: coaching experience and motivation for education as earning points (personal development- why do you attend in-person conferences/trainings/workshops?)

**Figure 4:**
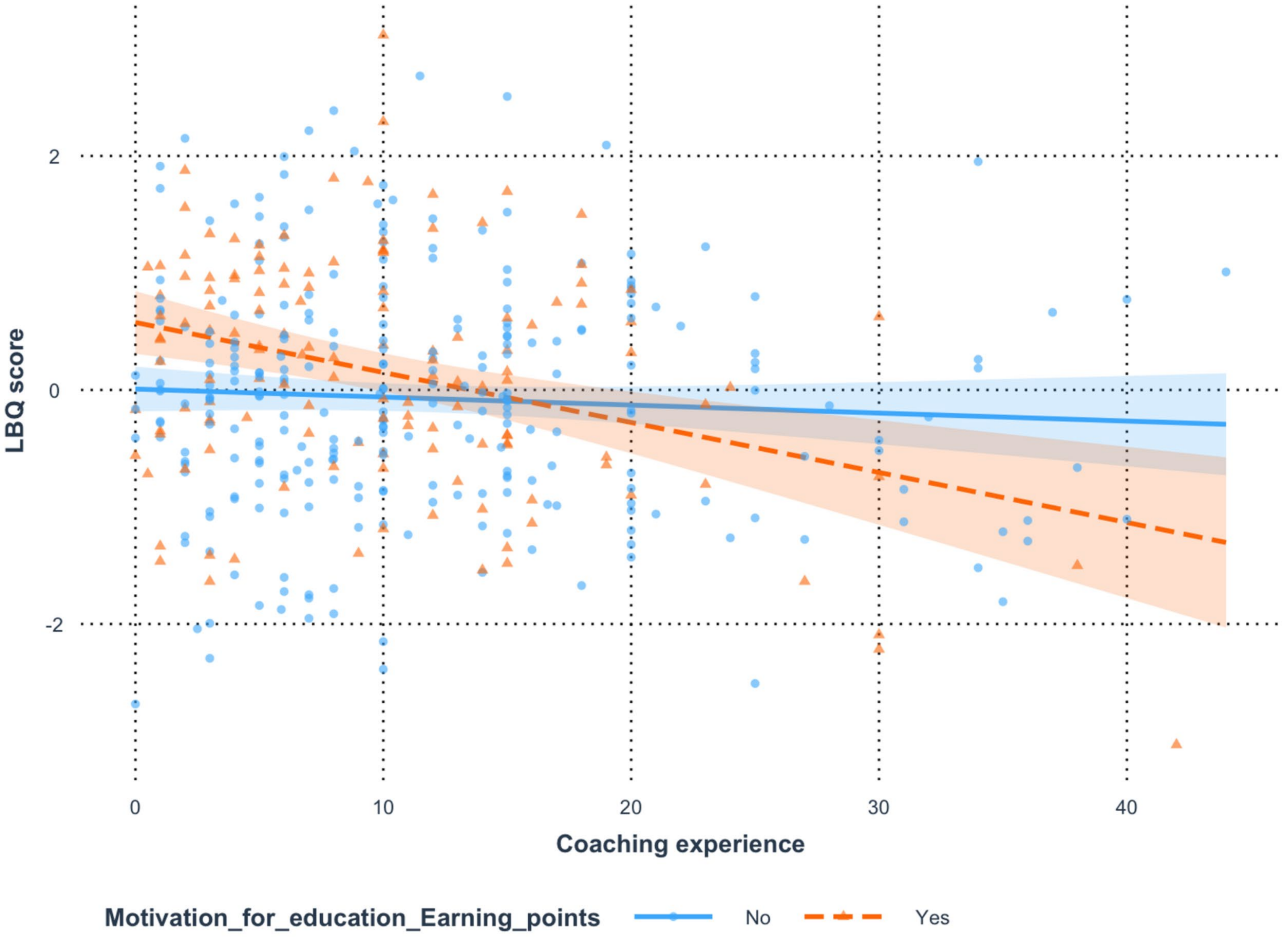
Relation between coaching experience and burnout moderated by motivation for education by earning points.

To analyze the results, an analysis of variance (Fisher, 1921) was conducted, which showed that Coaching experience did not have a significant effect on LBQ score [*F*(1, 408) = 1.07, *p* = 0.301, *η*^2^ = 0.00, $\eta_{p}^{2}$ = 0.00]. In contrast, Motivation for Education: Earning Points had a significant effect [*F*(1, 408) = 11.58, *p* < 0.001, *η*^2^ = 0.03, $\eta_{p}^{2}$ = 0.03]. Furthermore, the analysis of interaction effects in the tested model revealed a significant interaction between Coaching experience and Motivation for Education: Earning Points in predicting LBQ score [*F*(1, 408) = 8.22, *p* = 0.004, *η*^2^ = 0.02, $\eta_{p}^{2}$ = 0.02] (Figure 4). The model explained approximately 5.06% of the variance in LBQ score (adjusted: 4.36%). Figure 1 shows that the effect of Coaching experience on LBQ score differed between the “Points-Motivated” and “Non-Points-Motivated” groups. The magnitude of this difference was small, with Cohen’s *d* = 0.04, 95% CI [0.01, 0.06]. In the Non-Points-Motivated group, Coaching experience was not significantly related to LBQ score [*b* = −0.01, *t*(408) = −1.03, *p* = 0.301]. In contrast, in the Points-Motivated group, higher Coaching experience was significantly associated with lower LBQ score [*b* = −0.04, *t*(408) = −4.02, *p* < 0.001] (Tables 6, 7).

**Table 6:** Estimates of the significance of differences in the effect of the variable *coaching experience* on the variable *LBQ score* resulting from the moderating effect of the variable *motivation for education: earning points.*

Comparison	Difference	*t*	*p*	Cohen’s *d*	LCI	UCI
No*—*Yes	0.04	2.87	0.004	0.04	0.01	0.06

Compariso*n =* Statistical assessment of differences in the effect of the variable *Coaching experience* on the variable *LBQ score* between groups of the moderating variable *Motivation for education: Earning points*. Difference = Magnitude of the difference in score levels between the respective groups of the variable *Motivation for education: Earning points*. t = Student’s t statistic. Cohen’s d = Effect size coefficient *d* by Cohen. LCI = Lower confidence interval for Cohen’s *d*. UCI = Upper confidence interval for Cohen’s *d*. p = Statistical significance calculated without correction for pairwise multiple comparisons.

**Table 7:** Regression estimates of the effect of the variable *coaching experience* on the variable *LBQ score* in subgroups of the moderating variable *motivation for education: earning points.*

Group	b	s.e.	LCI	UCI	*t*	*p*
No	−0.01	0.01	−0.02	0.01	−1.03	0.301
Yes	−0.04	0.01	−0.06	−0.02	−4.02	< 0.001

b = Estimate of the effect of the variable *Coaching experience* on the variable *LBQ score* resulting from the moderating effect of the variable *Motivation for education: Earning points*. s.e. = Standard error of the estimate *b*. t = Student’s t statistic. LCI = Lower confidence interval for *b*. UCI = Upper confidence interval for *b*. p = Statistical significance calculated without correction for pairwise multiple comparisons.

### *Analysis 4*: number of teams managed currently (coach experience) and education level (personal development)

**Figure 5:**
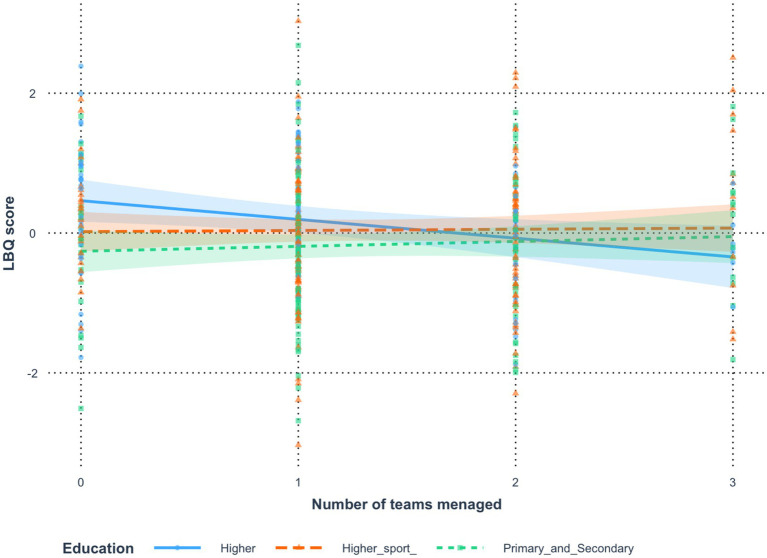
Relation between number of team managed and burnout moderated by motivation for education by earning points.

To analyze the results, an analysis of variance (Fisher, 1921) was conducted, which revealed that Number of teams managed had a significant effect on *LBQ score* [*F*(1, 406) = 6.25, *p* = 0.013, *η*^2^ = 0.01, $\eta_{p}^{2}$ = 0.02]. Similarly, Education showed a significant effect [*F*(2, 406) = 5.72, *p* = 0.004, *η*^2^ = 0.03, $\eta_{p}^{2}$ = 0.03]. Furthermore, the analysis of interaction effects indicated that Number of teams managed and Education interacted significantly in predicting *LBQ score* [*F*(2, 406) = 3.05, *p* = 0.048, *η*^2^ = 0.01, $\eta_{p}^{2}$ = 0.01]. The tested model explained approximately 3.40% of the variance in *LBQ score* (adjusted: 2.21%). *Post-hoc* comparisons of the moderating effect of Education showed that in the *Higher* education group, the effect of Number of teams managed on *LBQ score* was significantly different from that in the *Higher sport* group [*b* = −0.29, *t*(406) = −2.02, *p* = 0.044], with a small effect size (*d* = −0.29, 95% CI [−0.57, −0.01]). In the *Higher* group, more teams managed was significantly associated with lower *LBQ score* (*b* = −0.27, *t* = −2.50, *p* = 0.013), whereas in the *Higher sport* group, this relationship was not statistically significant (*b* = 0.02, *t* = 0.19, *p* = 0.852). Similarly, the *Higher* group differed significantly from the *Primary and Secondary* group [*b* = −0.34, *t*(406) = −2.30, *p* = 0.022], also with a small effect size (*d* = −0.34, 95% CI [−0.63, −0.05]). Again, the relationship was significant in the *Higher* group indicated that more teams managed was significantly associated with lower *LBQ score* (*b* = −0.27, *t* = −2.50, *p* = 0.013) but not significant in the *Primary and Secondary* group (*b* = 0.07, *t* = 0.70, *p* = 0.486). By contrast, the *Higher sport* and *Primary and Secondary* groups did not differ significantly (*b* = −0.05, *t*(406) = −0.39, *p* = 0.700, *d* = −0.05, 95% CI [−0.32, 0.22]). In both of these groups, the association between Number of teams managed and *LBQ score* was non-significant (*Higher sport*: *b* = 0.02, *t* = 0.19, *p* = 0.852; *Primary and Secondary*: *b* = 0.07, *t* = 0.70, *p* = 0.486). Figure 5 illustrates the moderating role of Education in the relationship between Number of teams managed and *LBQ score*, with shaded areas indicating 95% confidence intervals for the regression estimates (Tables 8, 9).

**Table 8:** Estimates of the significance of differences in the effect of the variable *number of teams managed* on the variable *LBQ score* resulting from the moderating effect of the variable *education.*

Comparison	Difference	*t*	*p*	Cohen’s *d*	LCI	UCI
Higher – Higher sport	−0.29	−2.02	0.044	−0.29	−0.57	−0.01
Higher*—*Primary and Secondary	−0.34	−2.30	0.022	−0.34	−0.63	−0.05
Higher sport*—*Primary and Secondary	−0.05	−0.39	0.700	−0.05	−0.32	0.22

Compariso*n =* Statistical assessment of differences in the effect of the variable *Number of teams managed* on the variable *LBQ score* across groups of the moderating variable *Education*. Difference = Magnitude of the difference in scores between respective groups of the variable *Education*.t = Student’s t statistic. Cohen’s d = Effect size coefficient *d* by Cohen. LCI = Lower confidence interval for Cohen’s *d*. UCI = Upper confidence interval for Cohen’s *d*. p = Statistical significance calculated without correction for pairwise multiple comparisons.

**Table 9:** Regression estimates of the effect of the variable *number of teams managed* on the variable *LBQ score* in subgroups of the moderating variable *education.*

Group	b	s.e.	LCI	UCI	*t*	*p*
Higher	−0.27	0.11	−0.48	−0.06	−2.50	0.013
Higher sport	0.02	0.09	−0.16	0.20	0.19	0.852
Primary and Secondary	0.07	0.10	−0.13	0.27	0.70	0.486

b = Estimate of the effect of the variable *Number of teams managed* on the variable *LBQ score* resulting from the moderating effect of the variable *Education*. s.e. = Standard error of the estimate *b*. t = Student’s t statistic. LCI = Lower confidence interval for *b*. UCI = Upper confidence interval for *b*. p = Statistical significance calculated without correction for pairwise multiple comparisons.

**Figure 6:**
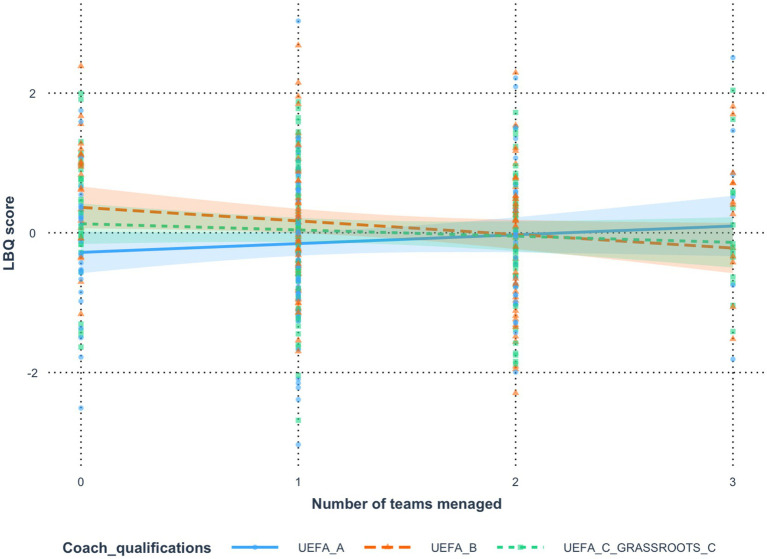
Relation between number of team managed and burnout moderated by motivation for education by earning points.

### *Analysis 5*: number of teams managed currently (coach experience) and coach qualifications (personal development)

The final analysis of variance showed that the Number of Teams Managed variable did not significantly influence the explanation of the LBQ score variable [*F*(1, 406) = 1.26, *p* = 0.263, *η*^2^ = 0.00, $\eta_{p}^{2}$ = 0.00], while the Coach Qualifications variable showed a significant influence [*F*(2, 406) = 4.35, *p* = 0.014, *η*^2^ = 0.02, $\eta_{p}^{2}$ = 0.02]. Analysis of the interaction effect in the tested model showed that the variables Number of Teams Managed and Coach Qualifications did not significantly interact with each other [*F*(2, 406) = 2.42, *p* = 0.090, *η*^2^ = 0.01, $\eta_{p}^{2}$ = 0.01]; however, it is worth noting that this effect was on the borderline of a statistical trend. Analysis of the R^2^ coefficient and its adjusted value showed that the tested model explained approximately 2.41% (1.20% after adjustment) of the variance in the results of the dependent variable LBQ score. The strength of the influence of the tested variables was assessed in terms of the value of the Cohen’s d statistic (Cohen, 1988). Figure 5 and the analysis of comparisons of the intensity of the effect of the Number of teams managed variable on the LBQ score variable showed that in UEFA group A it was statistically different than in UEFA group B *b* = 0.32; *t*(406) = 2.20; *p* = 0.029. The effect of the strength of differences between these groups was weak, Cohen’s d coefficient was d = 0.32; 95% CI [0.03; 0.61]. In UEFA group A, the effect of the Number of teams managed variable was not significantly associated with the change in the results of the LBQ score variable, however in UEFA group B an increase in the results of the Number of teams managed was associated with a significant decrease in the intensity of the LBQ score results, the results were, respectively, UEFA A b = 0.13; t = 1.16; *p* = 0.248 *Vs* UEFA b = −0.19; t = −2.00; *p* = 0.046. Further analysis of the effect of the number of managed teams on the LBQ result showed no significant differences between groups: UEFA B and UEFA C GRASSROOTS C [*b* = −0.11; *t*(406) = −0.78; *p* = 0.438] and UEFA A and UEFA C GRASSROOTS C [*b* = 0.21; *t*(406) = 1.49; *p* = 0.137] (Tables 10, 11).

**Table 10:** Estimates of the significance of differences in the effect of the variable *number of teams managed* on the variable *LBQ score* resulting from the moderating effect of the variable *coach qualifications.*

Comparison	Difference	*t*	*p*	Cohen’s *d*	LCI	UCI
UEFA A*—*UEFA B	0.32	2.20	0.029	0.32	0.03	0.61
UEFA A*—*UEFA C GRASSROOTS C	0.21	1.49	0.137	0.22	−0.07	0.50
UEFA B*—*UEFA C GRASSROOTS C	−0.11	−0.78	0.438	−0.11	−0.37	0.16

Compariso*n =* Statistical assessment of differences in the effect of the variable *Number of teams managed* on the variable *LBQ score* across groups of the moderating variable *Coach qualifications*. Difference = Magnitude of the difference in scores between respective groups of the variable *Coach qualifications*. t = Student’s t statistic. Cohen’s d = Effect size coefficient *d* by Cohen. LCI = Lower confidence interval for Cohen’s *d*. UCI = Upper confidence interval for Cohen’s *d*. p = Statistical significance calculated without correction for pairwise multiple comparisons.

**Table 11:** Regression estimates of the effect of the variable *number of teams managed* on the variable *LBQ score* in subgroups of the moderating variable *coach qualifications.*

Group	b	s.e.	LCI	UCI	*t*	*p*
UEFA A	0.13	0.11	−0.09	0.34	1.16	0.248
UEFA B	−0.19	0.10	−0.38	0.00	−2.00	0.046
UEFA C GRASSROOTS C	−0.09	0.09	−0.28	0.10	−0.94	0.348

b = Estimate of the effect of the variable *Number of teams managed* on the variable *LBQ score* resulting from the moderating effect of the variable *Coach qualifications*. s.e. = Standard error of the estimate *b*. t = Student’s t statistic. LCI = Lower confidence interval for *b*. UCI = Upper confidence interval for *b*. p = Statistical significance calculated without correction for pairwise multiple comparisons.

## Discussion

This study investigated how individual factors—such as age, coaching experience (coaching experience in years, number of teams managed), education (primary and secondary school, university, sport university), professional qualifications (UEFA A, UEFA B, UEFA C and GRASSROOTS C), and professional development (motivation for earning points for license renewal)—interact to predict burnout among soccer coaches. While looking at hypothesis 1 (*Age is associated with coaches’ burnout level and this relation is moderated by selected aspects of professional development and satisfaction with salary*), age was found to be negatively associated with soccer coaches’ burnout, particularly among those motivated by earning points for license renewal (personal development). This suggests that older soccer coaches, especially those driven by structured, extrinsic motives, may be better equipped to handle the emotional and physical demands of the profession. Their greater life and job experience may contribute to higher resilience and more effective coping strategies. From an evolutionary psychology perspective, resilience and coping can be understood as adaptive mechanisms that historically enhanced survival and social functioning (Buss, 2019). In modern contexts such as coaching, these evolved psychological traits may help individuals sustain motivation and manage stress, thereby reducing vulnerability to burnout. Moreover, these results can be interpreted through the lens of Self-Determination Theory (Deci and Ryan, 1985), which posits that as coaches focus on growth and develop their professional abilities, they fulfill the need for competence. According to SDT, this enhances wellbeing, which is negatively correlated with burnout (Ryan and Deci, 2000). Yet the modest effects observed in this study suggest that age is likely only one of several factors influencing burnout among coaches. The Satisfaction with salary did not moderate any of the association with measured variables as the authors expected. A comprehensive review by Belias et al. (2013) highlights that job satisfaction in coaches of different sport disciplines is influenced by factors such as salary, organizational commitment, and support. The study indicates that job burnout is strongly related to chronic stress and the persistent imbalance between demands and coping resources. Notably, the review suggests that coaches are characterized by high levels of job burnout, which vary based on sex, age, and work experience. This implies that salary satisfaction could potentially moderate the relationship between age or experience and burnout, although in our study the effects were not evident. A study conducted among competitive sports coaches in Sichuan Province, China, emphasizes that factors such as role, interpersonal relationships, and career development are closely related to occupational burnout. Importantly, the study identifies organizational support and coping strategies as mediators in the relationship between job pressure and burnout (Yu and Cheng, 2024).

Part of Hypothesis 2 (*Coaching experience is associated with coaches’ burnout and this relation is moderated by selected aspects of professional development and satisfaction with salary*) was also confirmed, with coaching experience inversely associated with coaches’ burnout, but primarily among those coaches who were motivated by earning education points (aspect of personal development). Again, looking from the perspective of SDT (1985), such motivation may reflect an internalization of professional development requirements (internalized extrinsic motivation), where the pursuit of education points supports the fulfillment of basic psychological needs—particularly competence and autonomy. In this context, structured goals like license renewal may serve not merely as external motives but as meaningful benchmarks that align with coaches’ personal values and growth. Ryan and Deci (Ryan and Deci, 2017) emphasize that when external goals, like earning education points, are internalized, they support the basic psychological needs of autonomy and competence, thereby promoting resilience against occupational stress. Nevertheless, although statistically significant, the effect size was weak, indicating limited practical significance.

Moreover, the relation between coaching experience—measured by the number of teams coached—and coach burnout was moderated by personal development such as educational background as well as coaching qualifications. Interestingly, soccer coaches with a general higher education degree seemed to benefit more from coaching multiple teams, possibly due to broader cognitive or organizational skills developed outside of sport-specific training, but also due to the way these experiences support the satisfaction of basic psychological needs, as proposed by SDT (Deci and Ryan, 1985). Moreover, individuals with higher education often show stronger tendencies toward intellectual curiosity and stimulation-seeking (Stumm et al., 2011), which can further enhance their ability to derive satisfaction and resilience from complex coaching roles. In contrast, those with sport-specific education or lower-level coaching licenses (e.g., UEFA C, GRASSROOTS C) appeared less resilient to managing multiple teams. However, given that the differences, although statistically significant, were of modest practical importance, broad generalizations should be avoided. Presumably the coaches with sport-specific education or lower-level licenses (e.g., UEFA C, GRASSROOTS C) may have fewer opportunities or motivational resources to interpret increased workload as a source of personal and professional growth. Thus, while more advanced qualifications may provide some advantage in coping with workload, the effect remains limited in scope.

Despite identifying several significant interaction effects, the models accounted for only a small proportion of the variance in coach burnout, and the observed effect sizes were consistently modest. These findings point to several important limitations. Most notably, the study did not include key psychological and contextual variables that are likely critical for understanding burnout among soccer coaches. Individual differences such as coping style, emotional intelligence, and personality traits (e.g., resilience, openness) were not assessed, yet have been shown in previous research to influence how coaches perceive and respond to stressors. Additionally, broader organizational and interpersonal factors—such as team climate, social support, and relationships with players and staff—were not captured in the current models. Prior studies have emphasized the importance of a positive team environment and high-quality coach–athlete relationships in promoting mental wellbeing and buffering against burnout in team sport contexts (Fan et al., 2023). These social dynamics may play a particularly important role in moderating the impact of workload and professional demands on psychological health. Moreover, the cross-sectional design of the study further limits the ability to draw causal inferences. Longitudinal data are needed to clarify the temporal direction of associations and to better understand how burnout develops and changes over time. Therefore, future research should adopt multi-level and longitudinal designs, incorporating both individual-level variables (e.g., psychological traits, motivational profiles) and systemic-level variables (e.g., organizational climate, leadership style). Observational and qualitative methods may also provide richer insights into the day-to-day realities of coaching and the nuanced social interactions that shape burnout risk. A more holistic approach could lead to the development of targeted interventions that better support coaches’ mental health and professional sustainability. Future studies should also expand current models by including psychological traits (e.g., resilience, emotional regulation, personality) as well as organizational climate and team dynamics, which may provide a more comprehensive understanding of burnout among soccer coaches.

From a practical perspective, the findings have important implications for soccer federations, clubs, and coach education providers. Tailored support should be offered to younger and less experienced coaches, especially those primarily motivated by license renewal. Integrating psychological skills training—such as stress management, emotional regulation, and time management—into coaching certification pathways could provide essential tools to help coaches cope with pressure. Additionally, federations might consider more flexible workload structures, particularly for lower-qualified coaches managing several teams, and emphasize the value of general higher education in developing versatile coaching skills. Federations should also consider offering a more flexible and differentiated coach education programs, adjusted to the needs of coaches with various license level. Given the modest effect sizes observed, these recommendations should be viewed as tentative, though they may still provide meaningful support when tailored to specific subgroups of coaches. Due to the cross-sectional design of the study, the implications should be interpreted with caution.

## Conclusion

Burnout primarily affects younger and less experienced coaches, particularly in the aspect of acquiring training points necessary for renewing their coaching licenses. However, this rigor does not diminish job satisfaction as coaches gain professional experience. A greater number of teams being managed contributes to professional burnout among coaches with higher specialized education. Therefore, in the educational practice of soccer coaches with little professional experience, it would be advisable to expand the existing training offers (conferences, workshops) to include other forms of acquiring training points, such as webinars, internships in clubs under the supervision of mentor coaches, authorship or co-authorship of methodological publications, presentations of their own achievements in the form of demonstration training sessions, lectures, or posters at methodological conferences. Conversely, for coaches with extensive professional experience and specialized education, conditions or material incentives should be created so that they can focus on coaching a single athlete group. The authors suggest a further need for research into professional burnout in soccer coaches.

## Data Availability

The raw data supporting the conclusions of this article will be made available by the authors, without undue reservation.

## References

[ref1] Agyapong B. Brett-MacLean P. Burback L. Agyapong V. I. O. Wei Y. (2023). Interventions to reduce stress and burnout among teachers: a scoping review. Int. J. Environ. Res. Public Health 20:5625. doi: 10.3390/ijerph20095625 37174145 PMC10178023

[ref2] Belias D. Koustelios A. Zournatzi E. Koutiva M. Sdrolias L. Ioanna B. (2013). Job satisfaction and job burnout of coaches: a review of the international literature. Int. J. Hum. Res. Manage. Res. 3, 27–38.

[ref3] Breiman L. (2001). Random forests. Mach. Learn. 45, 5–32. doi: 10.1023/A:1010933404324

[ref4] Buss D. M. (2019). Evolutionary psychology: The new science of the mind. 6th Edn. London: Routledge.

[ref5] Capone V. Joshanloo M. Park M. S.-A. (2019). Burnout, depression, efficacy beliefs, and work-related variables among school teachers. Int. J. Educ. Res. 95, 97–108. doi: 10.1016/j.ijer.2019.02.001

[ref6] Cohen J. (1988). Statistical power analysis for the behavioral sciences. 2nd Edn. London: Routledge.

[ref7] Deci E. L. Ryan R. M. (1985). “Conceptualizations of intrinsic motivation and self-determination” in Intrinsic motivation and self-determination in human behavior. eds. Deci E. L. Ryan R. M. (New York, NY: Springer US), 11–40.

[ref8] Fan F. Chen J. Chen Y. Li B. Guo L. Shi Y.. (2023). How relationship-maintenance strategies influence athlete burnout: mediating roles of coach–athlete relationship and basic psychological needs satisfaction. Front. Psychol. 13:1104143. doi: 10.3389/fpsyg.2022.1104143, PMID: 36698612 PMC9869133

[ref9] Fisher R. A. (1921). Studies in crop variation. I. An examination of the yield of dressed grain from Broadbalk. J. Agric. Sci. 11, 107–135. doi: 10.1017/S0021859600003750

[ref10] Gencay S. Gencay O. A. (2011). Burnout among judo coaches in Turkey. J. Occup. Health 53, 365–370. doi: 10.1539/joh.10-0064-FS, PMID: 21778661

[ref11] Gustafsson H. Kenttä G. Hassmén P. (2011). Athlete burnout: an integrated model and future research directions. Int. Rev. Sport Exerc. Psychol. 4, 3–24. doi: 10.1080/1750984X.2010.541927

[ref12] Hassmén P. Kenttä G. Hjälm S. Lundkvist E. Gustafsson H. (2019). Burnout symptoms and recovery processes in eight elite soccer coaches over 10 years. Int. J. Sports Sci. Coach. 14, 431–443. doi: 10.1177/1747954119851246

[ref13] Hjälm S. Kenttä G. Hassmén P. Gustafsson H. (2007). Burnout among elite soccer coaches. J. Sport Behav. 30, 415–427.

[ref14] Hryniewicz K. Milewska A. (2023). SZTOS: Automated Statistical Description System [System Zautomatyzowanego Tworzenia Opisu Statystycznego] (Wersja SZTOS) [Computer software]. Gdańsk.

[ref15] Hudson J. Davison G. Robinson P. (2013). Psychophysiological and stress responses to competition in team sport coaches: an exploratory study. Scand. J. Med. Sci. Sports 23, e279–e285. doi: 10.1111/sms.12075, PMID: 23662710

[ref16] Iancu A. E. Rusu A. Măroiu C. Păcurar R. Maricuțoiu L. P. (2018). The effectiveness of interventions aimed at reducing teacher burnout: a meta-analysis. Educ. Psychol. Rev. 30, 373–396. doi: 10.1007/s10648-017-9420-8

[ref17] Jaworowska A. (2014). LBQ: Link Burnout Questionnaire. [Kwestionariusz Wypalenia Zawodowego]. Warsaw: Psychological Tests Laboratory of the Polish Psychological Association. [Pracownia Testów Psychologicznych Polskiego Towarzystwa Psychologicznego].

[ref18] Jiahao L. Jing L. (2024). Examining the link between coach-athlete relationship and athlete burnout among college soccer players: the mediating role of training satisfaction. Front. Psychol. 15:1409609. doi: 10.3389/fpsyg.2024.1409609, PMID: 39165760 PMC11334079

[ref19] Koustelios A. (2010). Burnout among football coaches in Greece. Biol. Exercise 6, 5–12. doi: 10.4127/jbe.2010.0031

[ref9002] Lüdecke D. Ben-Shachar M. S. Patil I. Waggoner P. Makowski D. (2021). Performance: An R package for assessment, comparison and testing of statistical models. J. Open Source Aoftware 6.

[ref20] Lundkvist E. Gustafsson H. Hjälm S. Hassmén P. (2012). An interpretative phenomenological analysis of burnout and recovery in elite soccer coaches. Qual. Res. Sport Exerc. Health 4, 400–419. doi: 10.1080/2159676X.2012.693526

[ref21] Madigan D. J. Kim L. E. (2021). Does teacher burnout affect students? A systematic review of its association with academic achievement and student-reported outcomes. Int. J. Educ. Res. 105:101714. doi: 10.1016/j.ijer.2020.101714, PMID: 40885029

[ref9001] Maslach C. Jackson S. E. (1981). The measurement of experienced burnout. J Organiza. Behavior. 2, 99–113.

[ref22] Moen F. Kyhre K. Moldovan I. (2018). The effects of perceived autonomy on affect and burnout among sport coaches. Int. J. Sport Manag. 19, 339–359.

[ref23] Olusoga P. Bentzen M. Kenttä G. (2019). Coach burnout: a scoping review. Int. Sport Coach. J. 6, 42–62. doi: 10.1123/iscj.2017-0094

[ref24] Parker G. Tavella G. Hopcraft M. (2023). Exploring the validity of the Sydney burnout measure. Psychiatry Res. 326:Article 115271. doi: 10.1016/j.psychres.2023.115271 37290365

[ref25] Peterson R. A. (2021). Finding optimal normalizing transformations via bestNormalize. R J. 13, 310–329. doi: 10.32614/RJ-2021-041

[ref26] Poulsen M. Poulsen A. A. (2018). Optimising motivation and reducing burnout for radiation oncology trainees: a framework using self-determination theory. J. Med. Imaging Radiat. Oncol. 62, 684–691. doi: 10.1111/1754-9485.12725, PMID: 29718574

[ref27] Ryan R. M. Deci E. L. (2000). Self-determination theory and the facilitation of intrinsic motivation, social development, and wellbeing. Am. Psychol. 55, 68–78. doi: 10.1037/0003-066X.55.1.68 11392867

[ref28] Ryan R. M. Deci E. L. (2017). Self-determination theory: Basic psychological needs in motivation, development, and wellness: Guilford Press.

[ref29] Santinello M. (2007). Link Burnout Questionnaire manual. 1st Edn. London: Giunti Psychometrics.

[ref30] Seo E. Kim H. Sim Y. Ha M.-S. Kim U. Kim H. (2022). Burnout, presenteeism and workplace conditions of Korean taekwondo coaches of high-performance athletes. Int. J. Environ. Res. Public Health 19:5912. doi: 10.3390/ijerph19105912, PMID: 35627449 PMC9141872

[ref31] Shi H. (2024). The psychological mechanism of basic psychological need frustration affecting job burnout: a qualitative study from China. Front. Psychol. 15:1400441. doi: 10.3389/fpsyg.2024.1400441, PMID: 39534477 PMC11554459

[ref32] Siwiorek J. (2018). Model of professional burnout: theoretical perspective [Modele wypalenia zawodowego: ujęcie teoretyczne]. Society and Education. International Humanistic Studies. [Społeczeństwo i Edukacja. Międzynarodowe Studia Humanistyczne] 29, 261–274.

[ref33] Stekhoven D. J. Bühlmann P. (2012). MissForest—nonparametric missing value imputation for mixed-type data. Bioinformatics 28, 112–118. doi: 10.1093/bioinformatics/btr597, PMID: 22039212

[ref34] Stumm S. Hell B. Chamorro-Premuzic T. (2011). The hungry mind: intellectual curiosity is the third pillar of academic performance. Perspect. Psychol. Sci. 6, 574–588. doi: 10.1177/1745691611421204, PMID: 26168378

[ref35] Wickham H. (2016). ggplot2: Elegant graphics for data analysis. 2nd *Edn*. Cham: Springer International Publishing.

[ref36] Yu L. Cheng L. (2024). The work stress, occupational burnout, coping strategies and organizational support of elite sports coaches in Sichuan Province: the mediating role of organizational support. Front. Psychol. 15:1437234. doi: 10.3389/fpsyg.2024.1437234, PMID: 39171218 PMC11335728

